# Effects of Machined Surface Integrity on High-Temperature Low-Cycle Fatigue Life and Process Parameters Optimization of Turning Superalloy Inconel 718

**DOI:** 10.3390/ma14092428

**Published:** 2021-05-07

**Authors:** Xiaoping Ren, Zhanqiang Liu, Xiaoliang Liang, Pengcheng Cui

**Affiliations:** 1 School of Mechanical Engineering, Shandong University, Jinan 250061, China; renxiaoping@sdu.edu.cn (X.R.); sduliangxiaoliang@gmail.com (X.L.); sducuipengcheng@mail.sdu.edu.cn (P.C.); 2 Key Laboratory of High Efficiency and Clean Mechanical Manufacture of MOE and Key National Demonstration Center for Experimental Mechanical Engineering Education, Jinan 250061, China

**Keywords:** inconel 718, surface integrity, low-cycle fatigue life, parameters optimization

## Abstract

Machined surface integrity characteristics, including surface stresses, physical-mechanical properties and metallographic structures, play important roles in the fatigue performance of machined components. This work aimed at investigating the effects of machined surface integrity on high-temperature low-cycle fatigue life. The process parameters were optimized to obtain required surface integrity and fatigue life of the turning superalloy Inconel 718. The relationships between low-cycle fatigue life and machined surface integrity characterization parameters were established based on the low-cycle fatigue tests at a high temperature (650 °C). The sensitivities of turning process parameters to high-temperature low-cycle fatigue life were analyzed, and the optimization parameters were proposed with the goal of antifatigue manufacturing. Experimental results indicated that the impact order of the characterization parameters of machined surface integrity on the high-temperature low-cycle fatigue life were the degree of work hardening *R_HV_*, the residual stress in the cutting speed direction *S*_22_, the fatigue stress concentration factor *K_f_*, the degree of grain refinement *R_D_* and the residual stress in the feed direction *S*_33_. In the range of turning parameters of the experiments in this research, the cutting speeds could be 80~110 m/min, and the feed rate could be 0.10~0.12 mm/rev to achieve a longer high-temperature low-cycle fatigue life. The results can be used for guiding the fatigue-resistant manufacturing research of aeroengine superalloy turbine disks.

## 1. Introduction

Nickel-based superalloys have been widely used in aeroengine turbine disk manufacturing components due to their excellent comprehensive properties such as outstanding high-temperature strength, superior fatigue resistance and good fracture toughness [1,2,3]. Especially, Inconel 718 is a iron-nickel-chromium-based deformed nickel-based alloy, which still maintains good properties over a relatively large temperature range (−253~650 °C) [4,5]. Due to the harsh service environment, the manufacturing parts of aeroengines have high requirements for surface quality. Usually, the cutting process to remove excess material is performed after precision integral forging to achieve the final accuracy.

However, Inconel 718 is a typical difficult-to-machine material due to the characteristics of high thermal hardness, poor heat dissipation, and stickiness. The cutting process easily causes changes in the microstructure and physical-mechanical properties of the machined surface, which are different from the matrix structure [6]. Inconel 718 is very sensitive to stress concentration and strain rate, which is prone to low-cycle fatigue failure under complex service conditions of high temperature [7]. Moreover, most fatigue fractures start from the surface and subsurface of the manufacturing material. These changes in the surface properties of manufacturing components have a decisive influence on fatigue performance [8]. Exploring the influence laws of the machined surface integrity characteristics of Inconel 718 on the fatigue performance is an effective means to improve the fatigue resistance of manufacturing components.

Several scholars have carried out a series of studies on surface integrity after the machining process, and machined surface integrity evaluation was summarized. The International Society of Production Engineering (CIRP) Surface Integrity Cooperative Working Group [9] summarized the progress in cutting surface integrity tests and theories, which pointed out that advanced test technology and prediction model were the future development direction of surface integrity. M’Saoubi et al. [10] published a literature review on the surface integrity of stainless steel, nickel-based alloys and titanium alloys. They pointed out that the selection of the evaluation index for machined surface integrity depends on the workpiece materials and the application. They also suggested that the formation mechanisms and prediction of surface integrity were the key research directions. Guo et al. [11] summarized the research on the residual stress of the machined surface after cutting hardened steel, titanium alloy and nickel-based alloy. They pointed out that a multiscale simulation prediction model and verification were the challenges of surface residual stress. Thakur et al. [12] reviewed the research status of the surface integrity during cutting process of a nickel-based superalloy, and the machined surface damage, residual stress, microstructure, work hardening and its effects on fatigue performance were summarized. Liang et al. [3] reviewed the research status of tool wear effects on surface integrity during cutting of the difficult-to-cut materials such as titanium alloy and nickel-based superalloy, and the typical surface integrity characteristics include surface topography, microstructure changes and mechanical properties were summarized.

Many researchers investigated the effects of the geometric properties (surface roughness, microcracks) and physical-mechanical properties (hardness, residual stress) of the machined surface on the fatigue performance of nickel-based superalloy. However, inconsistent conclusions were drawn due to the different service conditions and load states targeted in the studies. Ardi et al. [13] found that the machined surface defects, surface roughness and work hardening were the dominant factors of the high-temperature low-cycle fatigue performance, while the research by Herbert et al. [14] suggested that the residual stress of the machined surface was the decisive factor. Barrie et al. [15] studied that the effect of the surface integrity induced by shot peening on the low-cycle fatigue life of the nickel-based alloy Udimet720, which indicated that shot peening could reduce the adverse effects of surface inclusions on fatigue life. Fielden et al. [16] studied the effects of surface integrity on the high-cycle fatigue life after milling Hastelloy C-2000, which found that work hardening and residual compressive stress improved the fatigue strength of manufacturing components. Moreover, Doremus et al. [17] studied the effects of different heat treatments on the low-cycle fatigue life of Inconel 718, which showed that the residual compressive stress retarded the crack growth in the first stage of fatigue crack growth. Chen et al. [18] investigated the effects of surface integrity induced by electrical discharge machining (EDM) on high-cycle fatigue life of Inconel 718. They found that the fatigue life of EDM specimens was reduced by about 30% compared with the polished specimen due to the microcracks and larger residual tensile stress on the workpiece surface.

To guide the antifatigue manufacturing of aeroengine superalloy turbine disks, this work investigated the effects of surface integrity on high-temperature low-cycle fatigue life and turning parameter optimization of Inconel 718. On the one hand, the response relationships between high-temperature low-cycle fatigue life and surface integrity characterization parameters were established by designing the high-temperature low-cycle fatigue test at 650 °C. On the other hand, the sensitivities of turning process parameters to high-temperature low-cycle fatigue life were analyzed, and the optimization parameters were proposed with the goal of antifatigue manufacturing.

## 2. Effects of Surface Integrity Characterization Parameters on Fatigue Life

**Figure 1:**
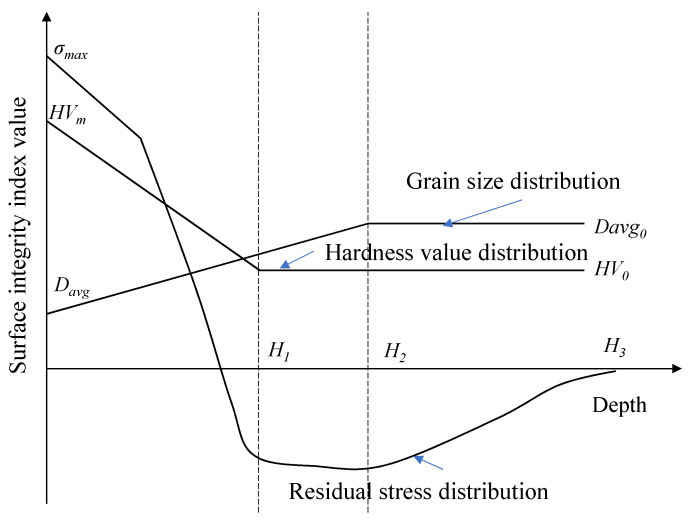
Gradient distribution of the surface integrity characterization parameters.

The machined surface integrity determines the service fatigue life of manufacturing parts by affecting the initiation and propagation of fatigue cracks. The manufacturing parts obtained by the cutting process, the microstructure and mechanical properties of the machined surface presented an obvious gradient distribution, which had the characteristics of inhomogeneity. As shown in Figure 1, surface integrity characteristics such as residual stress, hardness value and grains diameter size all presented the gradient distribution. The localized cyclic plastic deformation and fatigue crack initiation of nickel-based superalloys during the process of part fatigue failure was inseparable from the local stress concentration caused by the inhomogeneity structure of the machined surface [19].

In the cutting of the metamorphic layer, the distribution of residual stress with depth could be characterized by the exponential cosine decay function, as given by Equation (1) [20].

(1) $$\sigma (x)=C_{0}e^{-\frac{\varsigma w_{d}x}{\sqrt{1-\varsigma^{2}}}}\cos\left(w_{d}x+\varphi_{0}\right)$$

where *σ*(*x*) is the residual stress of the distance of position *x* from the machined surface, *C*_0_ is the residual stress amplitude constant, *ς* is the damping coefficient, *w_d_* is the damping frequency, and *φ* is the phase angle.

For simplification, the modification of hardness with depth is assumed to be linear. The function expression of the hardness *HV(x)* at the distance of depth *x* from the machined surface is given by Equation (2).

(2) $$HV\left(x\right)=\{\begin{array}{c} \left[R_{HV}-\frac{x}{H_{1}}\left(R_{HV}-1\right)\right]HV_{0}\\ HV_{0} \end{array}\begin{array}{c} x<H_{1}\\ x\geq H_{1} \end{array}$$

where *H*_1_ is the depth of the hardened layer. *R_HV_* is the ratio of the surface hardness to the substrate hardness, *R_HV_* = *HV_m_/HV*_0_. *R_HV_* reflects the hardening degree of the surface material after the turning process. *HV*_0_ was the initial microhardness value of the superalloy Inconel 718.

Following with the same simplification for hardness, the grain size distribution is simplified to a linear one, too. The function expression of the grain size *Davg(x)* at the depth *x* distance from the machined surface is given by Equation (3).

(3) $$D_{avg}\left(x\right)=\{\begin{array}{c} \left[R_{D}-\frac{x}{H_{2}}\left(R_{D}-1\right)\right]D_{avg}{}_{0}\\ D_{avg}{}_{0} \end{array}\begin{array}{c} x<H_{2}\\ x\geq H_{2} \end{array}$$

where *H*_2_ is the depth of the grain refinement layer, *R_D_* is the ratio of the surface grain size to the matrix grain size, and *D_avg_*_0_ is the initial grain size of Inconel 718.

In addition to the metallurgical physical and mechanical change characteristics of the machined surface layer, the influence of the surface geometry characteristics on the fatigue performance of the manufacturing parts is also important. The fatigue stress concentration factor (*K_f_*) was calculated by defining the stress concentration factor (*K_t_*) to evaluate the effects of the surface geometry on fatigue life of manufactured parts [21]. The relationship between the fatigue stress concentration factor *K_f_* and the stress concentration factor *K_t_* is given by Equation (4).

(4) $$K_{f}=1+q\left(K_{t}-1\right)$$

where *K_t_* is the stress concentration factor, and *q* is the notch sensitivity coefficient, which depends on the machined surface profile. The calculation is given by Equation (5).

(5) $$q=1/\left(1+\gamma /\bar{\rho }\right)$$

where $\bar{\rho }$ is the average value of the radius of curvature of the valley bottom of the machined surface profile and γ is the material constant, which is defined by the tensile strength *σ_u_*. The expression is shown in Equation (6).

(6) $$\gamma =0.025{\left(\frac{2070\mathrm{MPa}}{\sigma_{u}}\right)}^{1.8}\mathrm{mm}$$

*K_t_* is expressed as a function of the arithmetic mean deviation *R_a_*, peak-to-valley height *R_y_* and 10-point roughness *R_z_* and the radius of curvature of the bottom of the material surface contour [8], as given by Equation (7).

(7) $$K_{t}=1+n_{s}\left(\frac{R_{a}}{\rho }\right)\left(\frac{R_{y}}{R_{z}}\right)$$

where *n_s_* represents the stress state of the manufacturing part (*n_s_* = 1 represents the shear state; *n_s_* = 2 represents the tension state).

Considering the gradient distribution characteristics of machined surface index parameters, the main factors affecting the fatigue life of parts with gradient machined surface layers were the degree of work hardening *R_HV_*, the degree of grain refinement *R_D_*, the residual stress in the cutting speed direction *S*_22_, the residual stress in the feed direction *S*_33_, and the fatigue stress concentration factor *K_f_*. The fatigue life of parts was a function of the above influencing factors, as given by Equation (8).

(8) $$N_{f}=f\left(R_{HV},D_{avg},R_{D},\sigma_{sur},K_{f}\right)$$

## 3. Experiments

**Figure 2:**
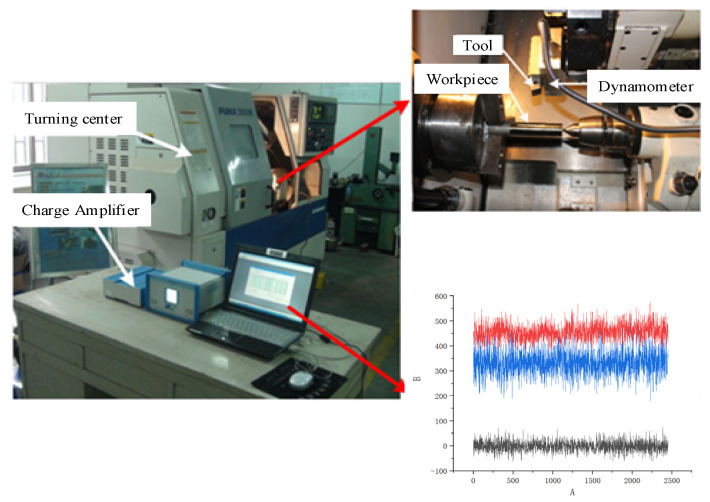
Set-up of machining experiments and test devices.

**Figure 3:**
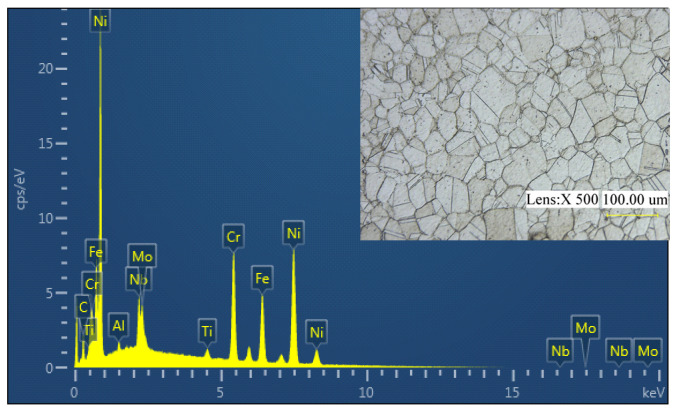
Chemical elements and phase analysis of Inconel 718.

As shown in Figure 2, the cutting experiment was implemented on Computer numerical control (CNC) turning lathe (PUMA 200M, Daewoo, Korea) in a dry environment. The cutting parameters were set as follows: cutting speed—*v* = 50, 70, 90, 110 m/min, feed rate—*f* = 0.1, 0.15, 0.2 mm/rev and depth of cut—*ap* = 0.2 mm. The workpiece material Inconel 718 used in the test was forged. The heat treatment system was a solid solution and aging treatment. The specific process was a solution treatment at 960 °C for 1 h, air cooling to room temperature, air cooling at 720 °C for 8 h aging treatment, then furnace cooling at a rate of 50 °C/h to 620 °C, which was held for 8 h and then air cooled. After heat treatment, the microhardness was 380 ± 10 HV. The chemical composition and microstructure of Inconel 718 are shown in Figure 3. The cutting tools were inserts of KC5525 with a nose radius of 0.8 mm supplied by Kennametal. The KC5525 insert was cemented carbide coated bys AlTiN with high hot hardness and good coating adhesion. The tool holder was SECO PSBNR252M12.

**Figure 4:**
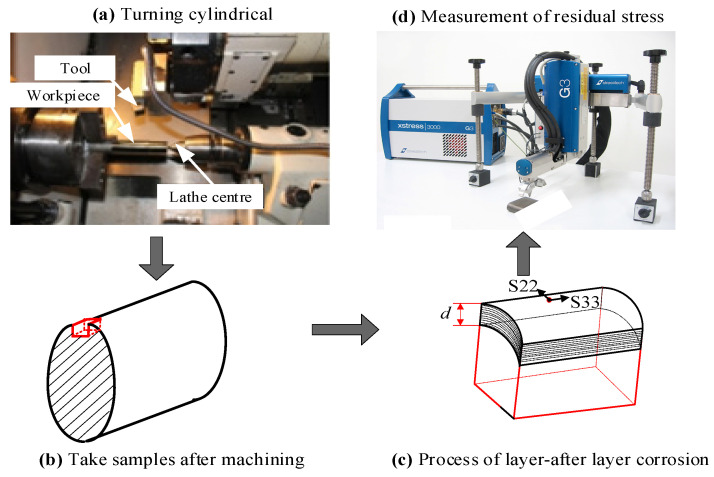
Procedure for residual stresses measurement.

After machining, the surface parameter measurements were carried out as follows. Surface topography features of the machined surface layer were characterized using laser scanning confocal microscopy (LSCM) (KEYENCE, Osaka, Japan). Microhardness measurements were obtained by Vickers microindentation hardness testing method with a diamond indenter subjected to a load of 50 g. The problem associated with microhardness measurement was its sensitivity to hard particles just below the workpiece surface. To overcome this problem, a series of measurements were taken repeatedly and an average microhardness value was obtained. The basic principle of measuring stresses for X-ray diffraction was used—the method of sin2ψ. The residual stresses in two-dimensional stress state were obtained, which were *S*_22_ (parallel to the cutting direction) and *S*_33_ (parallel to the feed direction), as shown in Figure 4. The microstructure of Inconel 718 before and after machining processing were examined using electron backscatter diffraction (EBSD, JOEL JSM-7800F, JEOL, Japan). For scanning electron microscope (SEM, JOEL JSM-7800F, JEOL, Japan) and EBSD examinations, samples after mechanical polishing (with Sic down to 2000 grit size) were electrolytically etched using a mixture of hydrochloric and nitric acids to reveal the microstructure.

**Figure 5:**
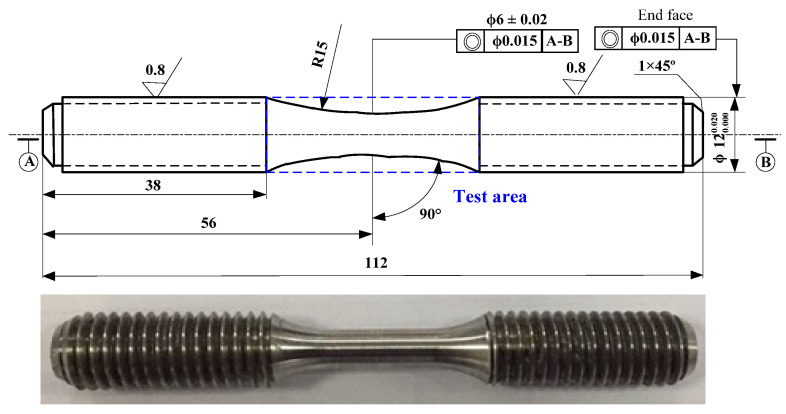
Geometric characteristics of the fatigue specimen.

The low-cycle fatigue life test of Inconel 718 at high temperature (650 °C) was carried out using round rod-shaped fatigue specimens. The geometric characteristics of the specimens were designed and manufactured according to GB/T15248-2008 (Axial Constant Amplitude Low-Cycle Fatigue Test Method for Metallic Materials) [22]. The dimensions of the fatigue specimen are shown in Figure 5. The total length of the manufacturing parts was 112 mm, and the radius of the clamping part was 6 mm. The radius of the working area was 3 mm, and the length was 18 mm. In order to avoid stress concentration, the smooth arc with a radius of 15 mm was used to connect the working part and clamping part.

The manufacturing sample was obtained by CNC turning. In order to ensure the consistency of the test conditions, the same batch of superalloy materials was selected and the same roughing process parameters were used for the peeling process. In order to analyze the effects of the surface integrity induced by different cutting speeds and feed rates on fatigue performance, 12 sets of fatigue specimens were designed, and the working part was turned according to the turning parameters of Inconel 718.

**Figure 6:**
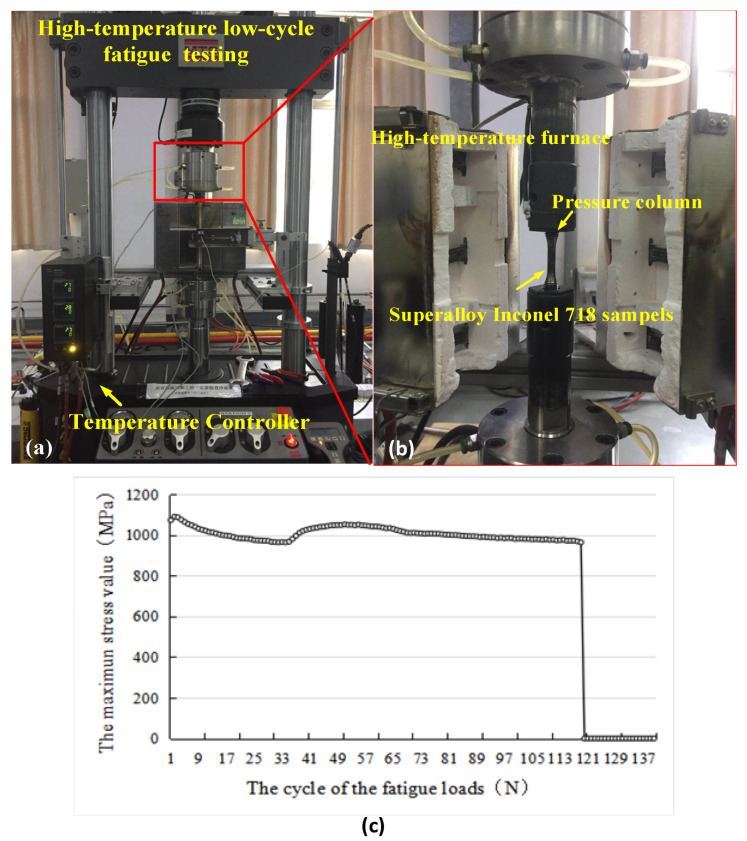
High-temperature low-cycle fatigue life test of Inconel 718. (**a**) Fatigue test machine; (**b**) clamping of sample during experiment; (**c**) the maximum stress changes with the number of cycles.

The equivalent stress borne by the turbine disk during service was about 900 MPa. The stress applied in the low-cycle fatigue tests was higher than 900 MPa. The low-cycle fatigue experiment was carried out on a fatigue testing machine (MTS370.10, MTS, USA), as shown in Figure 6. The loading parameters were set as 0.8% strain amplitude, stress ratio—R = −1, strain rate—0.4%, and strain waveform was a triangular wave; the loading stress under the condition was 1759 MPa. A high-temperature resistance furnace was used to heat specimens to 650 °C. The data of each condition were the average value of 3 samples. All specimens were loaded until fracture, and the cycle number at fracture was the low-cycle fatigue life of machined specimens.

The fracture morphology of specimens was observed using SEM (JOEL JSM-7800F, JEOL, Japan) to analyze the low-cycle fatigue crack initiation and propagation character. The low-cycle fatigue performances of Inconel 718 under different turning parameters were compared, and the effects of the surface layer gradient parameters on the fatigue life were revealed and the turning process parameters were further optimized.

## 4. Results and Discussions

### 4.1. Low-Cycle Fatigue Life and Fracture Morphology

Table 1 shows the low-cycle fatigue test results, which gives the response relationship between the low-cycle fatigue life *N_gf_*, turning process parameters (*v*, *f*, *a_p_*), and surface integrity indicators (*K_f_*, *S*_22_, *S*_33_, *R_HV_*, *R_D_*), respectively.

**Figure 7:**
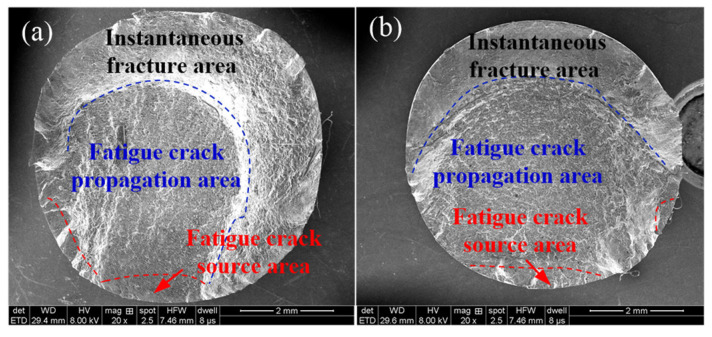
Macromorphology of high-temperature low-cycle fatigue fracture. (**a**) *v* = 70 m/min, *f* = 0.15 mm/rev, *a_p_* = 0.20 mm; (**b**) *v* = 50 m/min, *f* = 0.15 mm/rev, *a_p_* = 0.20 mm.

Fatigue fracture recorded the characteristics of each stage from the beginning to the end of the fatigue fracture process. Observing the fracture of the specimens was not only helpful to understand the mechanism of fatigue fracture, but also to analyze the response relationship of fatigue fracture to the machined metamorphic layer by comparing the structure and physical-mechanical properties. Figure 7 shows the macroscopic morphology of the low-cycle fatigue fracture of a typical Inconel 718 under high-temperature conditions. The fatigue fracture positions all appeared in the working section of the fatigue specimen and the initial direction of fracture was perpendicular to the direction of force. The low-cycle fatigue fracture was comprised of the fatigue crack source area, the fatigue crack propagation area and the instantaneous fracture area. The fatigue crack source area was bright silver-white, the fatigue crack propagation area was dark, and the instantaneous fracture area was dark gray.

Moreover, the fatigue crack source area was semielliptical. The fatigue crack growth area occupied most of the fracture area. The transient region had an uneven rough surface and a shear lip feature. Fatigue cracks on high-temperature fractures showed obvious multipoint origin characteristics, which was explained by the activation of multiple slip systems on the machined surface of the specimen when subjected to higher stress. These slip systems were more likely to cause multiple crack sources when they acted on defects of the machined surface layer.

**Figure 8:**
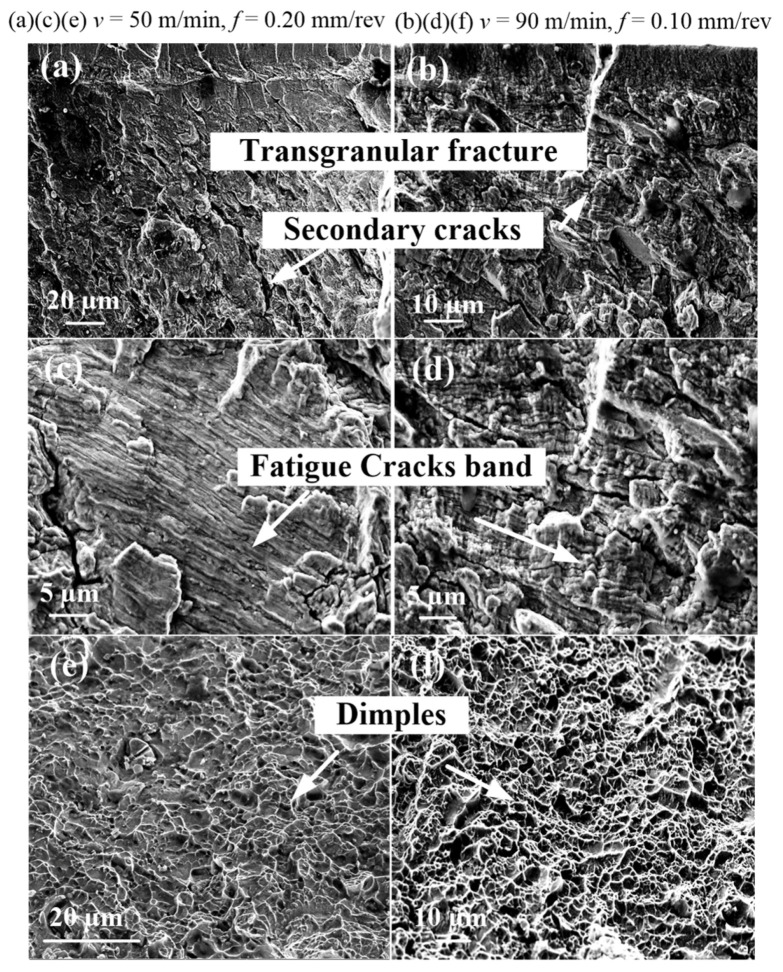
Low-cycle fatigue fracture morphology (**a**,**b**) source zone characteristics, (**c**,**d**) fatigue expansion zone characteristics, (**e**,**f**) transient fault zone characteristics. Note: (**a**,**c**,**e** high temperature *v* = 50 m/min, *f* = 0.20 mm/rev, *a_p_* = 0.20 mm; (**b**,**d**,**f**) high temperature *v* = 90 m/min, *f* = 0.10 mm/rev, *a_p_* = 0.20 mm).

Figure 8 shows the microscopic morphology of the low-cycle fatigue fracture of Inconel 718. Observing the morphology of the fatigue crack source area (Figure 8a,b), it was found that the fatigue cracks mainly originated on the machined surface of the low-cycle fatigue specimen with the transcrystalline mode, and existed in the initial stage of fatigue crack propagation. In addition, the multisource characteristics of high-temperature fatigue fracture showed the effects of temperature on the fracture strength of the machined metamorphic layer. The fracture strength decreased under high-temperature conditions, which showed the uniform distribution of the fracture source area. In the fatigue crack propagation area, clearer fatigue bands were observed. The spacing and shape of the fatigue band were related to the location. Near the fatigue crack source area, the fatigue bands were extremely dense and difficult to distinguish, as shown in Figure 8c,d. As the cracks grew, the fatigue bands gradually widened in the middle of the expansion. The fatigue instantaneous failure zone was characterized by dimple fracture, as shown in Figure 8e,f.

### 4.2. Effects of Surface Integrity on High-Temperature Low-Cycle Fatigue Life

#### 4.2.1. Effects of Residual Stress on Low-Cycle Fatigue Life

**Figure 9:**
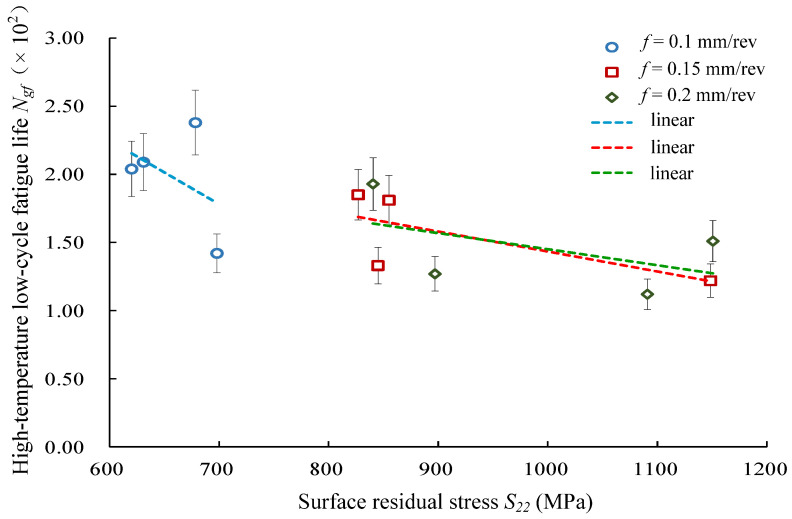
Effects of surface residual stress *S*_22_ (cutting speed direction) on low-cycle fatigue life.

**Figure 10:**
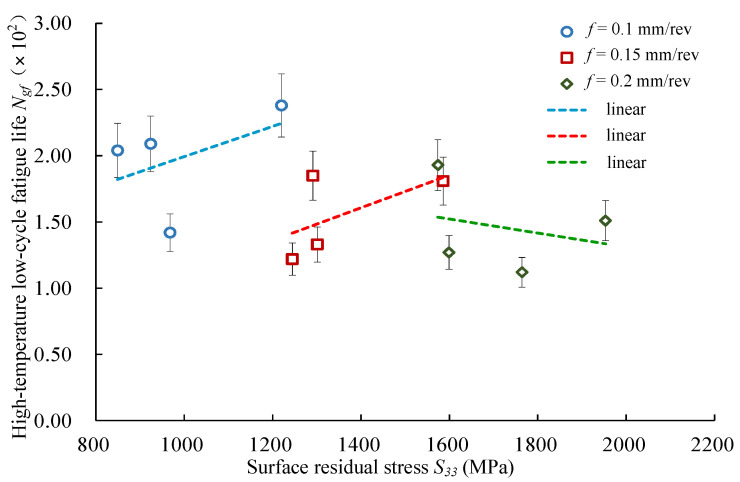
Effects of surface residual stress *S*_33_ (feeding direction) on low-cycle fatigue life.

Figure 9 presents the relationship between the surface residual stress *S*_22_ (cutting speed direction) and the high-temperature low-cycle fatigue life of Inconel 718. When the surface residual stress *S*_22_ increased, the fatigue life decreased at three different feed rates (*f* = 0.10 mm/rev, *f* = 0.15 mm/rev, *f* = 0.20 mm/rev). Figure 10 shows the relationship between the residual stress in the feed direction *S*_33_ and the high-temperature low-cycle fatigue life. It was seen that the fatigue life had an increasing trend with the surface residual stress *S*_33_ increasing at feed rates of *f* = 0.10 mm/rev and *f* = 0.15 mm/rev. However, the fatigue life presented a decreasing trend at the feed rate *f* = 0.20 mm/rev. The results indicate that the residual stress had a greater influence on fatigue performance when the feed rate was higher.

#### 4.2.2. Effects of Degree of Work Hardening on Low-Cycle Fatigue Life

**Figure 11:**
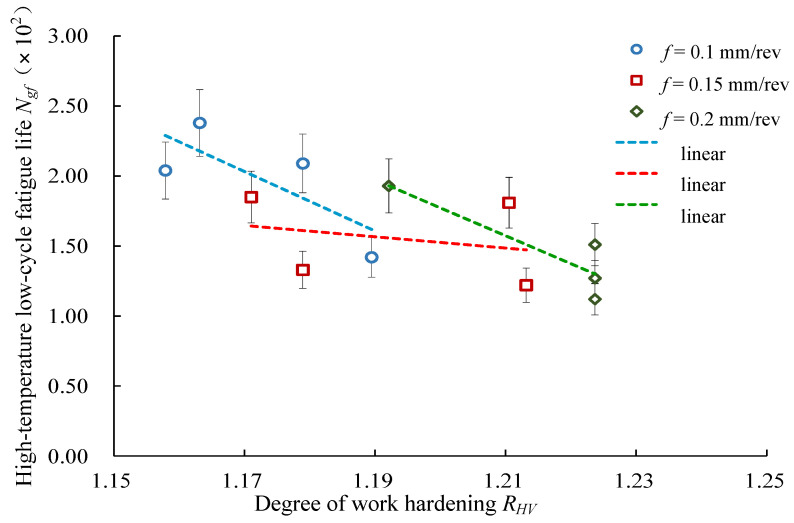
Effects of work hardening on low-cycle fatigue life.

Figure 11 presents the relationship between the high-temperature low-cycle fatigue life and degree of work hardening of the machined surface. As shown in Figure 11, as the degree of work hardening of the machined surface increased, the high-temperature low-cycle fatigue life decreased under the conditions of three different feed rates.

#### 4.2.3. Effects of Grain Refinement on Low-Cycle Fatigue Life

**Figure 12:**
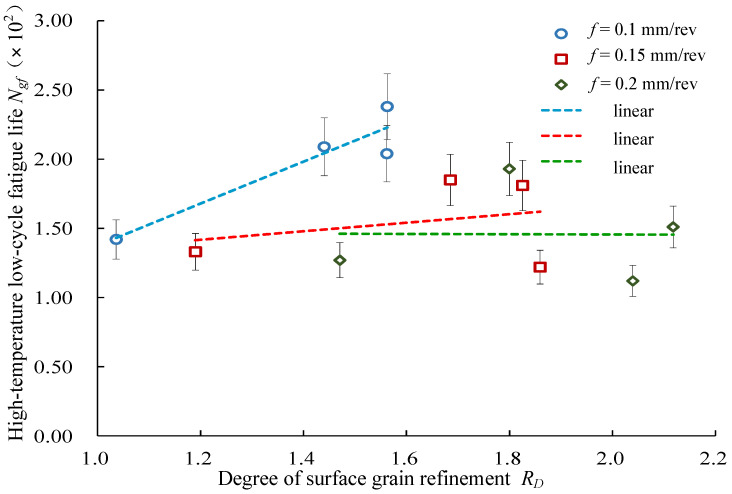
Effects of surface grain refinement on high-temperature low-cycle fatigue life.

Figure 12 shows the relationship between the degree of surface grain refinement of the turned Inconel 718 and the high-temperature low-cycle fatigue life. The high-temperature low-cycle fatigue life showed an increasing trend with an increase in the surface grain refinement. This was due to the fact that the fine grain structure had more grain boundary areas that hindered the movement of dislocations, which enabled more slip surfaces to move. The deformation was dispersed over more slip bands and was more uniform and the strain concentration was higher. Therefore, the chance of crack initiation was reduced. As a result, the fatigue life of coarse grains was lower than that of fine grain structures.

#### 4.2.4. Effects of Surface Fatigue Stress Concentration Coefficient on Low-Cycle Fatigue Life

**Figure 13:**
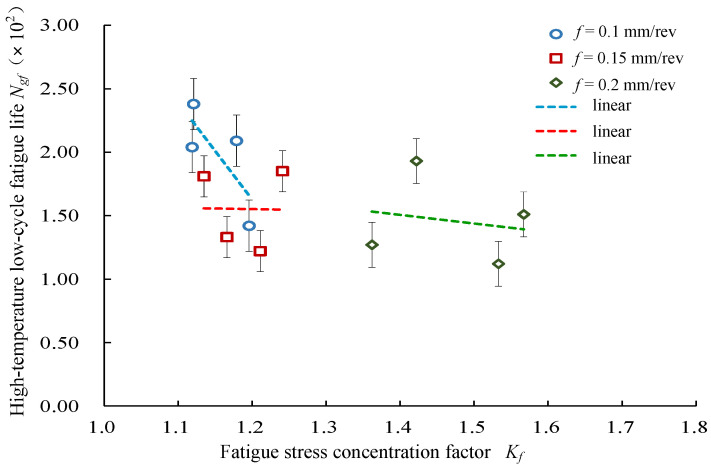
Effects of fatigue stress concentration factor *K_f_* on high-temperature low-cycle fatigue life.

Figure 13 presents the response relationship between the fatigue stress concentration factor *K_f_* and the high-temperature low-cycle fatigue life. It was seen that the high-temperature low-cycle fatigue life changed with the fatigue stress concentration coefficient *K_f_* under three different feed conditions, which showed that the high-temperature low-cycle fatigue life decreased with the increase in the fatigue stress concentration coefficient.

An empirical formula was established between the high-temperature low-cycle fatigue life and the fatigue stress concentration factor *K_f_*, the degree of work hardening *R_HV_*, the residual stress in the cutting speed direction *S*_22_, the residual stress in the feed direction *S*_33_, and the degree of grain refinement *R_D_*, as given by Equation (9).

(9) $$N_{gf}=k_{f}{}^{-1.14}\cdot R_{HV}{}^{-4.35}\cdot S_{22}{}^{-1.14}\cdot S_{33}{}^{0.383}\cdot R_{D}{}^{0.702}$$

It can be seen from Equation (9) that the order of the characterization parameters of machined surface integrity for the high-temperature low-cycle fatigue life is the degree of work hardening *R_HV_*, the residual stress in the cutting speed direction *S*_22_, the fatigue stress concentration factor *K_f_*, the degree of grain refinement *R_D_* and the residual stress in the feed direction *S*_33_.

### 4.3. Optimization of Inconel 718 Antifatigue Turning Process Parameters

The effects of machined surface integrity on fatigue performance were investigated to optimize the turning process parameters in order to obtain better surface integrity and achieve a long fatigue life. Based on the above analysis, the effects of the turning process parameters on fatigue life were analyzed, and the parameters were further optimized to improve the high-temperature low-cycle fatigue life.

**Figure 14:**
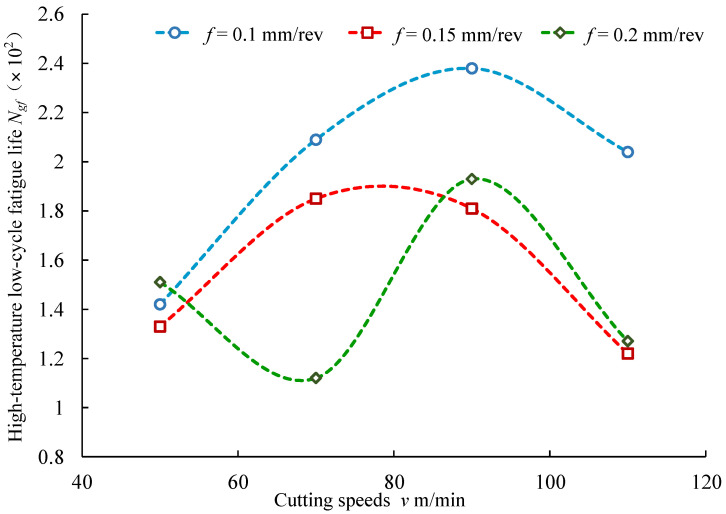
Effects of cutting speeds on high-temperature low-cycle fatigue life.

Figure 14 presents the effects of cutting speeds on the high-temperature low-cycle fatigue life of the superalloy Inconel 718. As shown in Figure 14, when the feed rates were *f* = 0.1 mm/rev and *f* = 0.15 mm/rev, the fatigue life firstly increased and then decreased with the increase in cutting speeds. Under the condition of *f* = 0.2 mm/rev, the fatigue life first decreased slightly, then increased and finally decreased with the increase in cutting speeds.

**Figure 15:**
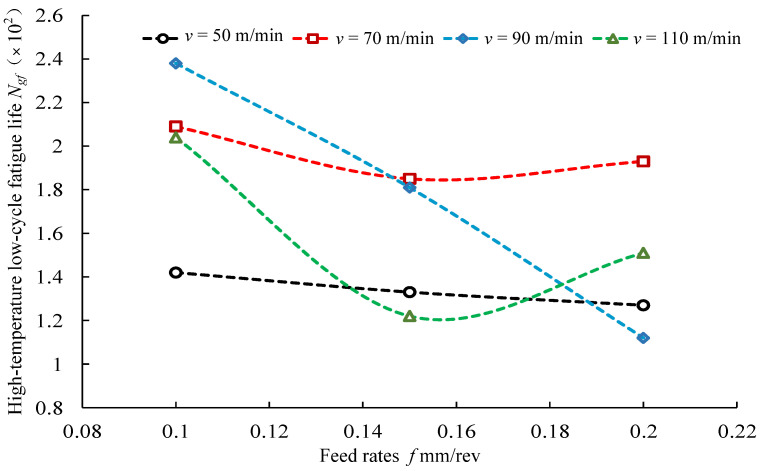
Effects of feed rates on high-temperature low-cycle fatigue life.

The effects of feed rates on high-temperature low-cycle fatigue life are shown in Figure 15. At lower cutting speeds, *v* = 50 m/min and *v* = 70 m/min, the high-temperature low-cycle fatigue life first increased and then decreased with the increase in feed rates from *f* = 0.10 mm/rev to *f* = 0.20 mm/rev. At higher cutting speeds, *v* = 90 m/min and *v* = 110 m/min, the high-temperature low-cycle fatigue life decreased with the increase in feed rates from *f* = 0.10 mm/rev to *f* = 0.20 mm/rev.

To further optimize the turning process parameters, the regression relationship model between the high-temperature low-cycle fatigue life of the superalloy Inconel 718 and turning process parameters are shown in Equation (10).

(10) $$N_{gf}=31\cdot v^{0.192}\cdot f^{-0.461}$$

**Figure 16:**
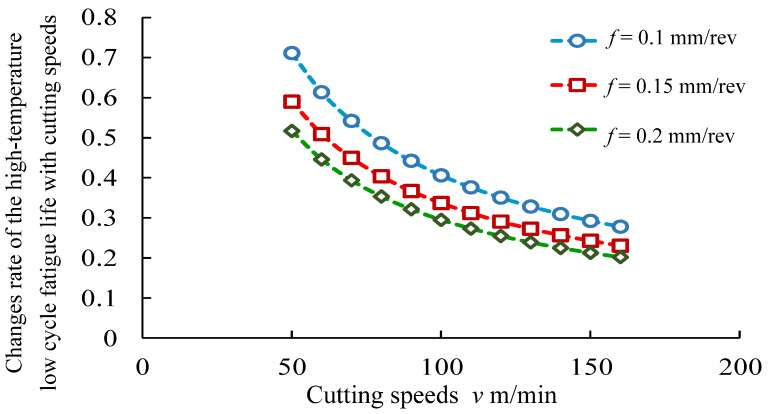
Sensitivity analysis of high-temperature low-cycle fatigue life to cutting speeds.

**Figure 17:**
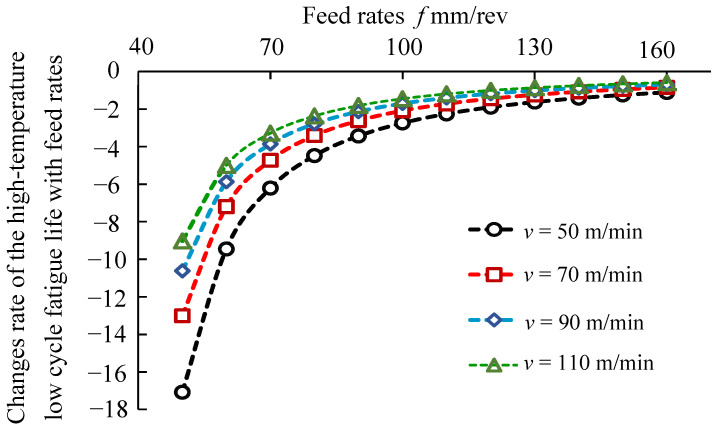
Sensitivity analysis of high-temperature low-cycle fatigue life to feed rates.

Based on the derivation of the cutting speeds and feed rates in Equation (10), the analysis of the sensitivity of high-temperature low-cycle fatigue life to cutting speeds and feed rates was carried out, as shown in Figure 16 and Figure 17. Figure 16 presents the analysis of sensitivity of high-temperature low-cycle fatigue life to cutting speeds. It was seen that the change rates of the effects of cutting speeds on high-temperature low-cycle fatigue life were in the range of 0.20~0.72. Figure 17 presents the sensitivity analysis of high-temperature low-cycle fatigue life to feed rates. The results indicated that the change rates of the high-temperature low-cycle fatigue life to the feed rates were in the range of −17.1~−0.59.

Within the range of the turning process parameters in this work (cutting speeds 50~110 m/min, feed rates 0.10~0.20 mm/rev), it was advisable to select a higher cutting speed (80~110 m/min) and a lower feed rate (0.10~0.12 mm/rev) to obtain a better high-temperature low-cycle fatigue life.

## 5. Conclusions

To guide the antifatigue manufacturing of aeroengine superalloy turbine disks, this work investigated the effects of surface integrity on high-temperature low-cycle fatigue life and turning parameters optimization of Inconel 718. The main conclusions are listed as follows.

(1) The fatigue crack growth area occupied most of the fracture area. The fatigue instantaneous failure zone was characterized by dimple fracture. The transient region had an uneven rough surface and a shear lip feature. Fatigue cracks on high-temperature fractures showed obvious multipoint origin characteristics. In addition, the multisource characteristics of high-temperature fatigue fracture showed the effects of temperature on the fracture strength of the machined metamorphic layer.
(2) With the increase in the surface residual stress *S*_22_, the fatigue life decreased at three different feed rates. The fatigue life displayed an increasing trend with the surface residual stress *S*_33_ increasing at the feed rates *f* = 0.10 mm/rev and *f* = 0.15 mm/rev, but presented a decreasing trend at the feed rate *f* = 0.20 mm/rev. The low-cycle fatigue life showed the different degrees of decreasing trend as the degree of work hardening of the machined surface increased. The low-cycle fatigue life showed an increasing trend with the increase in the surface grain refinement and presented a decreasing trend with the increase in the fatigue stress concentration coefficient.
(3) The order of the characterization parameters of machined surface integrity on the high-temperature low-cycle fatigue life was the degree of work hardening *R_HV_*, the residual stress in the cutting speed direction *S*_22_, the fatigue stress concentration factor *K_f_*, the degree of grain refinement *R_D_* and the residual stress in the feed direction *S*_33_.
(4) In the range of turning parameters of this experiment, the cutting speeds should be 80~110 m/min, and the feed rate should be 0.10~0.12 mm/rev to obtain higher high-temperature low-cycle fatigue life.

## Figures and Tables

**Table 1:** Relationship between the low-cycle fatigue life, turning process parameters, and surface integrity indicators.

Specimens No.	Turning Process Parameters			Surface Integrity Indicators					Low-Cycle Fatigue Life
	*v* (m/min)	*f* (mm/rev)	*a_p_* (mm)	*K_f_*	*S*_22_ (MPa)	*S*_33_ (MPa)	*R_HV_*	*R_D_* (mm)	*N_gf_* (× 10^2^)
1	50	0.10	0.20	1.196	698	968.0	1.189	1.04	1.420
2	50	0.15	0.20	1.166	845	1301.0	1.179	1.19	1.330
3	50	0.20	0.20	1.362	897	1599.0	1.224	1.47	1.270
4	70	0.10	0.20	1.179	631	924.0	1.179	1.44	2.090
5	70	0.15	0.20	1.241	827	1291.0	1.171	1.69	1.850
6	70	0.20	0.20	1.422	840.5	1574.0	1.192	1.80	1.930
7	90	0.10	0.20	1.121	678.4	1219.7	1.163	1.56	2.380
8	90	0.15	0.20	1.135	855	1585.6	1.211	1.83	1.810
9	90	0.20	0.20	1.533	1090.9	1764.3	1.244	2.04	1.120
10	110	0.10	0.20	1.119	620.1	849.0	1.158	1.56	2.040
11	110	0.15	0.20	1.211	1148.5	1244.4	1.213	1.86	1.220
12	110	0.20	0.20	1.567	1150.7	1953.1	1.224	2.12	1.510

## Data Availability

The data presented in this study are available on request from the corresponding author.
